# South African hypertension practice guideline 2014

**DOI:** 10.5830/CVJA-2014-062

**Published:** 2014

**Authors:** YK Seedat, BL Rayner, Yosuf Veriava

**Affiliations:** Department of Medicine, University of KwaZulu-Natal, Durban, South Africa; Department of Medicine, University of Cape Town, Cape Town, South Africa; Department of Medicine, University of Cape Town, Cape Town, South Africa

**Keywords:** South Africa, hypertension, guideline

## Abstract

**Outcomes:**

Extensive data from many randomised, controlled trials have shown the benefit of treating hypertension (HTN). The target blood pressure (BP) for antihypertensive management is systolic < 140 mmHg and diastolic < 90 mmHg, with minimal or no drug side effects. Lower targets are no longer recommended. The reduction of BP in the elderly should be achieved gradually over one month. Co-existent cardiovascular (CV) risk factors should also be controlled.

**Benefits:**

Reduction in risk of stroke, cardiac failure, chronic kidney disease and coronary artery disease.

**Recommendations:**

Correct BP measurement procedure is described. Evaluation of cardiovascular risk factors and recommendations for antihypertensive therapy are stipulated. Lifestyle modification and patient education are cornerstones of management. The major indications, precautions and contra-indications are listed for each antihypertensive drug recommended. Drug therapy for the patient with uncomplicated HTN is either mono- or combination therapy with a low-dose diuretic, calcium channel blocker (CCB) and an ACE inhibitor (ACEI) or angiotensin receptor blocker (ARB). Combination therapy should be considered *ab initio* if the BP is ≥ 160/100 mmHg. In black patients, either a diuretic and/or a CCB is recommended initially because the response rate is better compared to an ACEI. In resistant hypertension, add an alpha-blocker, spironolactone, vasodilator or β-blocker.

**Validity:**

The guideline was developed by the Southern African Hypertension Society 2014©.

## Abstract

This is the sixth hypertension guideline published by the Southern African Hypertension Society (SAHS). Currently 30.4% of the adult population have hypertension (HTN),^1^ necessitating a simplified approach to assessment and treatment, which reflects realistic objectives that can be implemented by medical practitioners, nurse practitioners and pharmacists to diminish the impact of HTN and related cardiovascular disease (CVD) risk in this country. For full details on management not contained in this document please refer to the more detailed hypertension guideline 2011.^2^

## Objective

The objective of this guideline was to promote evidencebased, accessible and comprehensive management of HTN by healthcare professionals in the public and private sectors. Applicable HTN and CVD treatment and prevention guidelines were reviewed as well as HTN trials reporting clinical end-points, including those with individuals with important co-morbidities such as diabetes mellitus and chronic kidney disease.^3^-^9^

## Definition and grading of hypertension

HTN is defined as a persistent elevation of office blood pressure (BP) ≥ 140/90 mmHg (Table 1). The optimal BP is a value < 130/85 mmHg. High normal is BP levels from 130–139 mmHg systolic and 85–89 mmHg diastolic. This high-normal group of subjects is at higher CV risk and is also at risk of developing HTN, but does not require drug treatment.^10^ HTN is stratified into three grades depending on severity, which is useful in defining the approach to treatment.

**Table 1:** Definitions and classification of office BP (mmHg). Adapted from ref 9

*Stage*	*Systolic BP (mmHg)*	*Diastolic BP (mmHg)*
Normal	< 120	< 80
Optimal	120–129	80–84
High normal	130–139	85–89
Grade 1	140–159	90–99
Grade 2	160–179	100–109
Grade 3	≥ 180	≥ 110
Isolated systolic	≥ 140	< 90

BP should be categorised into the highest level of BP whether systolic or diastolic.

## Measurement of blood pressure

BP measurement is a vital clinical sign that is poorly performed by all healthcare professional categories. These recommendations apply to both clinic and self-measurement of BP. Failure to follow these guidelines leads to significant errors in BP measurement. BP should be recorded using an approved and calibrated electronic device or mercury sphygmomanometer (Table 2). Repeat measurements should be performed on at least three separate occasions within four weeks unless BP is ≥ 180/110 mmHg.

**Table 2:** Recommendations for blood pressure measurement

Allow patient to sit for 3–5 minutes before commencing measurement
The SBP should be first estimated by palpation to avoid missing the auscultatory gap
Take two readings 1–2 minutes apart. If consecutive readings differ by > 5 mm, take additional readings
At initial consultation measure BP in both arms, and if discrepant use the higher arm for future estimations
The patient should be seated, back supported, arm bared and arm supported at heart level
Patients should not have smoked, ingested caffeine-containing beverages or food in previous 30 min
An appropriate size cuff should be used: a standard cuff (12 cm) for a normal arm and a larger cuff (15 cm) for an arm with a mid-upper circumference > 33 cm (the bladder within the cuff should encircle 80% of the arm)
Measure BP after 1 and 3 minutes of standing at first consultation in the elderly, diabetics and in patients where orthostatic hypotension is common
When adopting the auscultatory measurement use Korotkoff 1 and V (disappearance) to identify SBP and DBP respectively
Take repeated measurements in patients with atrial fibrillation and other arthythmias to improve accuracy

## Self- and ambulatory measurement of BP

Self BP measurement (SBPM) and ambulatory BP measurement (ABPM) are recommended in selected circumstances and target groups:^11^

• suspected white-coat HTN (higher readings in the office compared with outside) or masked HTN (normal readings in office but higher outside)
• to facilitate diagnosis of HTN
• to guide antihypertensive medication, especially in high-risk groups, e.g. elderly, diabetics
• refractory HTN
• to improve compliance with treatment (SBPM only).

Masked HTN should be suspected if, despite a normal BP in the clinic, there is evidence of target-organ damage.

All devices used for SBPM and ABPM should be properly validated in accordance with the following independent websites: or http://afssaps.sante.fr.

In general, only upper-arm devices are recommended, but these are unsuitable in patients with sustained arrhythmias. For SBPM the patient should take two early morning and two late afternoon/early evening readings over five to seven days, and after discarding the first day readings, the average of all the remaining readings is calculated.

Wrist devices are recommended only in patients whose arms are too obese to apply an upper arm cuff. The wrist device needs to be held at heart level when readings are taken.

The advantages of SBPM measurement are an improved assessment of drug effects, the detection of causal relationships between adverse events and blood pressure response, and possibly, improved compliance. The disadvantages relate to increased patient anxiety and the risk of self-medication.

ABPM provides the most accurate method to diagnose HTN, assess BP control and predict outcome.^12^ Twenty-four-hour ABPM in patients with a raised clinic BP reduces misdiagnosis and saves costs.^13^ Additional costs of ABPM were counterbalanced by cost savings from better-targeted treatment. It can also assess nocturnal BP control and BP variability, which are important predictors of adverse outcome. However the assessment is limited by access to ABPM equipment, particularly in the public sector, and impracticalities of regular 24-hour ABPM monitoring.

The appropriate cut-off levels for diagnosis of HTN by SBPM and ABPM are listed in Table 3.^11^

**Table 3:** Definitions of hypertension by different methods of BP measurement

	*Office*	*Automated office*	*Self*	*Ambulatory*
Predicts outcome	+	++	++	+++
Initial diagnosis	Yes	Yes	Yes	Yes
Cut-off BP (mmHg)	140/90	Mean 135/85	135/85	Mean day 135/85 Mean night 120/70 Mean night 120/70
Evaluation of treatment	Yes	Yes	Yes	Limited, but valuable
Assess diurnal variation	No	No	No	Yes

## Automated office BP measurement

Despite efforts to promote proper techniques in manual BP measurement, it remains poorly performed. Automated office BP measurement offers a practical solution to overcome the effects of poor measurement, bias and white coating.^14^ It is more predictive of 24-hour ABPM and target-organ damage than manual office BP measurement. Six readings are taken at two-minute intervals in a quiet room. The initial reading is discarded and the remaining five are averaged. The appropriate cut-off level for HTN is 135/85 mmHg.^14^

## CVD risk stratification

The principle of assessing and managing multiple major risk factors for CVD is endorsed. However, because the practical problems in implementing previous recommendations based on the European Society of HTN (ESH) and the European Society of Cardiology (ESC) HTN guidelines, it has been decided to use a modification of this approach.^9^

Once the diagnosis of HTN is established, patients with BP ≥ 160/100 mmHg should commence drug therapy and lifestyle modification. Patients with stage 1 HTN should receive lifestyle modification for three to six months unless they are stratified as high risk by the following criteria: three or more major risk factors, diabetes, target-organ damage or complications of HTN (Table 4).

**Table 4:** Major risk factors, target-organ damage (TOD ) and complications. Adapted from the ESH/ESC guidelines^9^

*Major risk factors*	*TOD*	*Complications*
• Levels of systolic and diastolic BP	• LVH: based on ECG	• Coronary heart disease
• Smoking	–– Sokolow-Lyons > 35 mm	• Heart failure
• Dyslipidaemia:	–– R in aVL > 11 mm	• Chronic kidney disease:
–– total cholesterol > 5.1 mmol/l, OR	–– Cornel > 2 440 (mm/ms)	–– macroalbuminuria > 30 mg/mmol
–– LDL > 3 mmol/l, OR	• Microalbuminuria: albumin creatine ratio 3–30 mg/mmol preferably spot morning urine and eGFR > 60 ml/min	–– OR eGFR < 60 ml/min
–– HDL men < 1 and women < 1.2 mmol/l		• Stroke or TIA
• Diabetes mellitus		• Peripheral arterial disease
• Men > 55 years		• Advanced retinopathy:
• Women > 65 years		–– haemorrhages OR
• Family history of early onset of CVD:		–– exudates
–– Men aged < 55 years		–– papilloedema
–– Women aged < 65 years		
• Waist circumference: abdominal obesity:		
–– Men ≥ 102 cm		
–– Women ≥ 88 cm		
–– The exceptions are South Asians and Chinese: men: > 90 cm and women: > 80 cm.		

## Routine baseline investigations

Table 5 lists recommended routine basic investigations. The tests are performed at baseline and annually unless abnormal. Abnormal results must be repeated as clinically indicated.

**Table 5:** Routine investigations

*Test*	*Comment*
Height, weight, BMI	Ideal BMI < 25 kg/m^2^, overweight 25–30 kg/m^2^, obese > 30 kg/m^2^
Waist circumference	Men < 102 cm; women < 88 cm. South Asians and Chinese: men < 90 cm and women < 80 cm
Electrolytes	Low potassium may indicate primary aldosteronism, or effects of diuretics
ECG	S in V1 plus R in V5 or V6 > 35 mm or R in aVL > 11 mm or Cornel product (R in aVL + S in V3 + 6 in females) × QRS duration > 2 440 (mm/ms)
Echocardiogram (if indicated and facilities available)	LVH: men > 115 g/m^2^ and women > 95 g/m^2^
Fasting glucose	Consider HBA1c or GTT if impaired fasting glucose (6.1–7.1 mmol/l)
Cholesterol	If total cholesterol > 5.1 mmol/l – fasting lipogram
Creatinine	Calculate eGFR
Uric acid	High uric acid is relative contraindication to diuretics
Dipsticks urine	If abnormal, urine microscopy and protein estimation

## Goals of treatment

There has been considerable controversy about BP goals and SAHS accepts that to simplify management, a universal goal of antihypertensive treatment is < 140/90 mmHg regardless of CV risk and underlying co-morbidities.^5^ The only exception is that in patients over 80 years of age, therapy should be initiated if SBP is > 160 mmHg and the goal is between 140 and 150 mmHg, based on the HYVET study in which the majority of patients received indapamide and the ACEI perindopril.^15^

SAHS does not support the JNC-8 committee recommendations of a goal BP < 150/90 mmHg for persons over 60 years without diabetes and CKD, as (1) increasing the target will probably reduce the intensity of antihypertensive treatment in a large population at high risk for cardiovascular disease, (2) the evidence supporting increasing the SBP target from 140 to 150 mmHg in persons aged 60 years or older was insufficient, (3) the higher SBP goal in individuals aged 60 years or older may reverse the decades-long decline in CVD, especially stroke mortality.^8^,^16^

It is also essential to control hyperlipidaemia and diabetes through lifestyle and drug therapy, according to the Society for Endocrine Metabolism Diabetes of South Africa and South African Heart Association/Lipid and Atherosclerosis Society of Southern Africa guidelines, respectively.^17^,^18^ Aspirin should not be routinely prescribed to hypertensives (especially if BP is not controlled),^19^ and should mainly be used for secondary prevention of CVD (transient ischaemic attack, stroke, myocardial infarction).

## Management of hypertension

**Fig. 1.**
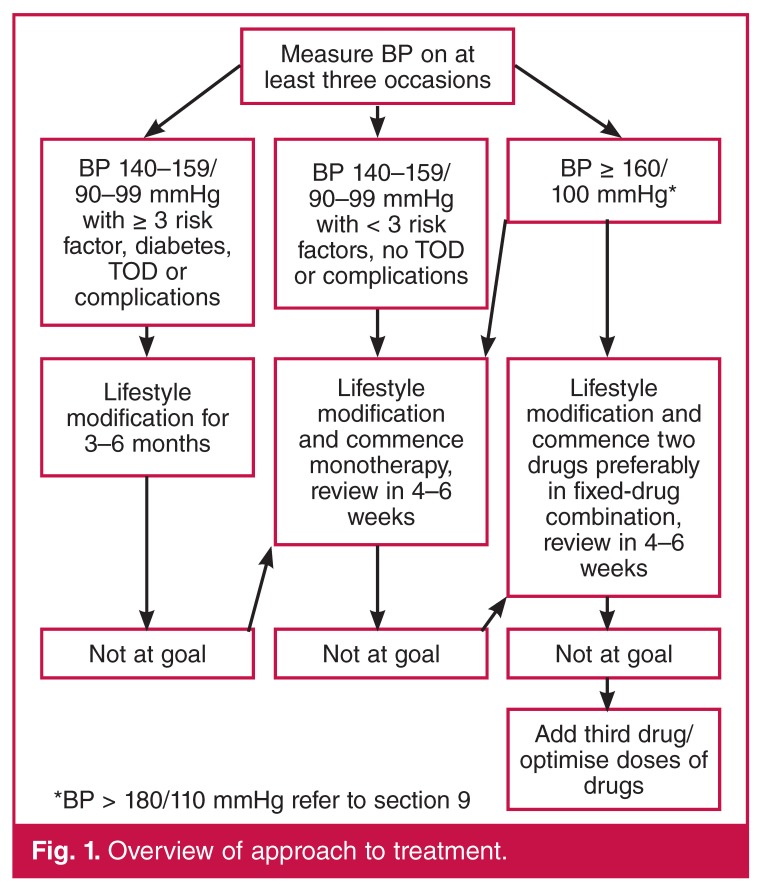
Overview of approach to treatment.

All patients with HTN should receive lifestyle counselling as outlined in Table 6, and this is the cornerstone of management. The approach to drug treatment is outlined in Fig. 1. *If the SBP is ≥ 180 mmHg or the DBP is ≥ 110 mmHg then refer to section 8 on severe (grade 3) HTN, as this section does not apply.*

**Table 6:** Recommended lifestyle changes

*Modification*	*Recommendation*	*Approx ↓ SBP (mmHg)*
Weight reduction	BMI 18.5–24.9 kg/m2	5–20 per 10 kg
Dash diet	↓ saturated fat and total fat, ↑ fruit and vegetables	8–14
Dietary Na+	< 100 mmol or 6 g NaCl/day	2–8
Physical activity	Brisk walking for 30 minutes per day most days	4–9
Moderation of alcohol	No more than two drinks per day	2–4
Tobacco	Complete cessation	–

Before choosing an antihypertensive agent, allow for considerations based on the cost of the various drug classes, patient-related factors, conditions favouring use and contraindications, complications and target-organ damage (TOD) (Tables 4, 7).

**Table 7:** Indications and contra-indications for the major classes of antihypertensive drugs. Adapted from the ESC/ESH guidelines^9^

		*Contra-indications*	
*Class*	*Conditions favouring the use*	*Compelling*	*Possible*
Diuretics (thiazide; thiazide-like)	• Heart failure( HF)	• Gout	• Pregnancy
	• Elderly hypertensives		• β-blockers (especially atenolol)
	• Isolated systolic HTN (ISH)		
	• Hypertensives of African origin		
Diuretics (loop)	• Renal insufficiency		• Pregnancy
	• HF		
Diuretics (anti-aldosterone)	• HF	• Renal failure	
	• Post-myocardial infarction	• Hyperkalaemia	
	• Resistant hypertension		
CCB (dihydropyridine)	• Elderly patients		• Tachyarrhythmias
	• ISH		• HF especially with reduced ejection fraction
	• Angina pectoris		
	• Peripheral vascular disease		
	• Carotid atherosclerosis		
	• Pregnancy		
CCB non-dihydropyridine (verapamil, diltiazem)	• Angina pectoris	• AV block (grade 2 or 3)	• Constipation (verapamil)
	• Carotid atherosclerosis	• HF	
	• Supraventricular tachycardia		
ACEI	• HF	• Pregnancy	
	• LV dysfunction	• Hyperkalaemia	
	• Post-myocardial infarction	• Bilateral renal artery stenosis	
	• Non-diabetic nephropathy	• Angioneurotic oedema (more common in blacks than in Caucasians)	
	• Type 1 diabetic nephropathy		
	• Prevention of diabetic microalbuminuria		
	• Proteinuria		
ARB	• Type 2 diabetic nephropathy	• Pregnancy	
	• Type 2 diabetic microalbuminuria	• Hyperkalaemia	
	• Proteinuria	• Bilateral renal artery stenosis	
	• LVH		
	• ACEI cough or intolerance		
β-blockers	• Angina pectoris	• Asthma	• Peripheral vascular disease
	• Post-myocardial infarction	• Chronic obstructive pulmonary disease	• Bradycardia
	• HF (carvedilol, metoprololol, bisoprolol, nebivolol only)	• AV block (grade 2 or 3)	• Glucose intolerance
	• Tachyarrhythmias	• Pregnancy (atenolol)	• Metabolic syndrome
			• Athletes and physically active patients
			• Non-dihydropyridine CCBs (verapamil, diltiazem)

**Fig. 2.**
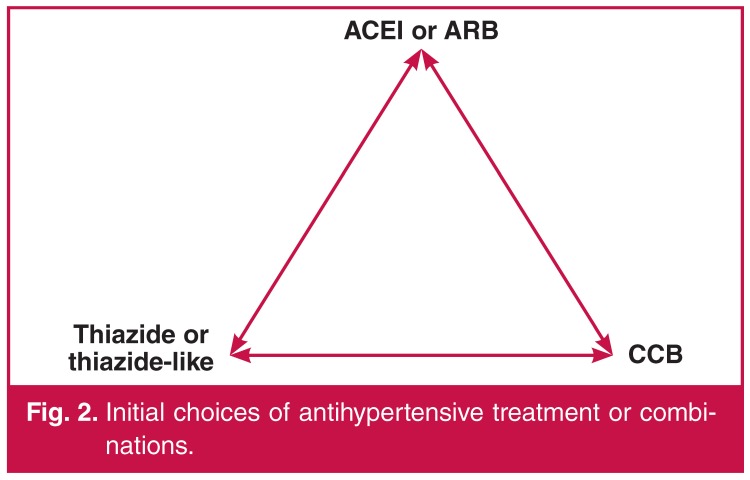
Initial choices of antihypertensive treatment or combinations.

In otherwise uncomplicated primary HTN, the initial first choice of antihypertensive drug is a diuretic (thiazide-like or thiazide), ACEI or ARB, and/or CCB used as mono- or combination therapy (Fig. 2). Combination therapy should be considered if clinically appropriate *ab initio* if BP is ≥ 160/100 mmHg (Fig. 1) as this is associated with better clinical outcomes and earlier achievement of goal BP.^20^,^21^ Fixed-drug combinations are preferred because of better patient adherence and control of BP.^22^ A treatment algorithm is outlined in Fig. 1 if the goal is not reached after initial treatment.

In black hypertensive patients a diuretic and/or a CCB is recommended.^23^ Beta-blockers should generally be avoided in combination with diuretics as first-line therapy because of predisposition to diabetes,^9^ but this may not apply to highly selective beta-blockers. Beta-blockers may also be considered if there is intolerance to one of the first-line drugs. Loop diuretics such as furosemide should not be used because of their short duration of hypotensive activity of about six hours, unless there is evidence of chronic kidney disease (CKD) with estimated glomerular filtration rate (GFR) < 45 ml/min.

## Management of severe hypertension

Patients with severe HTN (grade 3; BP ≥ 180/110 mmHg) may fall into one of three categories, which determine the urgency of their treatment. Patients should be managed or referred to the appropriate level of care and caregiver in accordance with local resources. Sustained, severe HTN requires immediate drug therapy and lifestyle modification, and close follow up.

## Asymptomatic severe hypertension

These patients are asymptomatic but have severe HTN without evidence of progressive TOD or complications. The patient must be kept in the care setting and BP measurement repeated after resting for one hour. If still elevated at the same level, commence oral therapy using two first-line drugs. Follow up within a week or earlier, with escalation of treatment as needed. Early referral is advised if BP is not controlled within two to four weeks.

## Hypertensive urgencies and emergencies^24^

While not common, hypertensive emergencies and urgencies are likely to be encountered by all clinicians because of the high prevalence of chronic HTN. It is essential that all professionals are familiar with treatment. There is a paucity of information from well-conducted studies on the outcomes of various antihypertensive drugs and BP-lowering strategies.

## *Hypertensive urgency*^25^

This level of HTN is symptomatic, usually with severe headache, shortness of breath and oedema. There are no immediate life-threatening neurological, renal, eye or cardiac complications, such as are seen in hypertensive emergencies. Ideally, all patients with hypertensive urgency should be treated in hospital.

Commence treatment with two oral agents and aim to lower the diastolic BP to 100 mmHg slowly over 48 to 72 hours. This BP lowering can be achieved with the use of: (1) long-acting CCBs; (2) ACEI, initially used in very low doses, but avoid if there is severe hyponatraemia (serum Na < 130 mmol/l indicates hyper-reninaemia and BP may fall dramatically with ACEI); (3) β- blockers; and (4) diuretics.

## Hypertensive emergency

A hypertensive emergency is severe, often acute elevation of BP associated with acute and ongoing organ damage to the kidneys, brain, heart, eyes (grade 3 or 4 retinopathy) or vascular system. These patients need rapid (within minutes to a few hours) lowering of BP to safe levels. Hospitalisation is ideally in an intensive care unit (ICU) with experienced staff and modern facilities for monitoring. If an ICU is unavailable, the patient may be closely monitored and treated in the ward.

Intravenous antihypertensive therapy, tailored to the specific type of emergency, has become the standard of care. Labetalol, nitroprusside or nitroglycerin are the preferred intravenous agents. Overzealous lowering of BP may result in stroke. A 25% reduction in BP is recommended in the first 24 hours. Oral therapy is instituted once the BP is more stable. Although most adult patients with a hypertensive emergency will have BP > 220/130 mmHg, it may also be seen at modest BP elevations; for example, in a previously normotensive woman during pregnancy (eclampsia) or in the setting of acute glomerulonephritis, especially in children.

Severe HTN associated with ischaemic stroke and intracerebral haemorrhage should be managed according to the recommendations of the Neurological Association of South Africa.^26^ Great caution should be exercised in lowering BP after an ischaemic stroke due to the risk of extending the ischaemic penumbra.

## Resistant hypertension

HTN that remains > 140/90 mmHg despite the use of three antihypertensive drugs in a rational combination at full doses and including a diuretic (hydrochlorothiazide 25 mg or indapamide 2.5 mg) is known as resistant HTN. Common causes of resistant HTN are listed in Table 8.

**Table 8:** Causes of resistant hypertension in South Africa

Non-adherence to therapy	• Instructions not understood
	• Side effects
	• Cost of medication and/or cost of attending at healthcare centre
	• Lack of consistent and continuous primary care
	• Inconvenient and chaotic dosing schedules
	• Organic brain syndrome (e.g. memory deficit)
Volume overload	• Excess salt intake
	• Inadequate diuretic therapy
	• Progressive renal damage (nephrosclerosis)
Associated conditions	• Smoking
	• Increasing obesity
	• Sleep apnoea
	• Insulin resistance/hyperinsulinaemia
	• Ethanol intake of more than 30 g (three standard drinks) daily
	• Anxiety-induced hyperventilation or panic attacks
	• Chronic pain
	• Intense vasoconstriction (Raynaud’s phenomenon), arteritis
Identifiable causes of hypertension	• Chronic kidney disease
	• Renovascular disease
	• Primary aldosteronism
	• Coarctation
	• Cushing’s syndrome
	• Phaeochromocytoma
Pseudoresistance	• ‘Whitecoat hypertension’ or office elevations
	• Pseudohypertension in older patients
	• Use of regular cuff in obese patients
Drug-related causes	• Doses too low
	• Wrong type of diuretic
	• Inappropriate combinations
	• Rapid inactivation (e.g. hydralazine)
Drug actions and interactions	• Non-steroidal anti-inflammatory drugs (NSAIDs)
	• Sympathomimetics: nasal decongestants, appetite suppressants
	• Cocaine, Tik and other recreational drugs
	• Oral contraceptives
	• Adrenal steroids
	• Liquorice (as may be found in chewing tobacco)
	• Cyclosporine, tacrolimus, erythropoietin
	• Antidepressants (monoamine oxidase inhibitors, tricyclics)

The therapeutic plan must include measures to ensure adherence to therapy and lifestyle changes. Unsuspected causes of secondary HTN are less common, but need to be considered based on history, examination and special investigations. It is essential to exclude pseudo-resistance by performing SBPM or 24-hour ABPM. Referral to a specialist is often indicated for a patient with resistant HTN.

Once the issues relating to lifestyle, adherence to therapy, white coating, etc. outlined in Table 7 have been satisfactorily managed, then consideration should be given to the addition of the fourth- and fifth-line drug. Currently spironolactone (25–50 mg only) with careful monitoring of serum potassium, beta-blockers and/or long-acting doxazasin is recommended.^27^,^28^ Other choices include direct-acting vasodilators (hydralazine, minoxidil), or centrally acting drugs (methyldopa, moxonidine, reserpine).

Initial studies of renal denervation in patients with resistant HTN showed very promising results.^29^,^30^ The recent publication of the Simplicity 3 study showing no significant effect on BP compared to sham procedure, the place of renal denervation in the treatment of resistant HTN remains to be established and is not supported by this guideline.^31^

## Special considerations for hypertension in certain populations

## Blacks and Asians

Blacks are more prone to complications of stroke, heart failure and renal failure, while the incidence of coronary heart disease, although increasing in frequency, is less common compared with that in whites and Asians.^32^ The prevalence of diabetes mellitus and the metabolic syndrome is higher in Asians compared to other racial groups.^33^

Compared to whites, blacks respond poorly to ACEI and β-blockers as monotherapy, but this difference disappears once these drugs are combined with diuretics. Overall, CCBs show the most consistent response in blacks compared to other classes of drugs used as monotherapy.^23^,^34^ However there is a higher incidence of angioedema in blacks treated with an ACEI.^35^

## Hypertension in children and adolescents^36^,^37^

HTN in children is an important issue beyond the scope of this guideline. In adolescents, the HTN is increasingly linked to obesity and affects up to 10% of people between the ages of 15 and 25 years.^38^ The international trend of poor diet and lack of exercise in children is leading to an epidemic of obesity, with the early onset of HTN and even type 2 diabetes. The early recognition of HTN in these adolescents will be an important motivation for both children and parents to institute important lifestyle changes.

## HIV/AIDS

There are an estimated 5.8 million people living with HIV in South Africa. The co-existence of HIV with HTN and diabetes is increasing, and patients should be screened for associated glomerulonephritis.^39^ Prolonged highly active antiretroviral therapy (HAART) is associated with a higher prevalence of systolic HPT,^40^ and it is essential that BP is monitored in patients receiving HAART.

Two of the three major classes of antiretroviral drug, the protease inhibitors and the non-nucleoside reverse transcriptase inhibitors, are involved in many drug interactions by inhibiting or inducing the key hepatic enzyme system, cytochrome P450. CCBs are the major class of antihypertensives affected by such drug interactions, leading to inhibition or induction of their metabolism.^41^,^42^ This results in either an enhanced or loss of antihypertensive efficacy.

## Disclaimer

This national clinical guideline is a reference and educational document. The SAHS accepts no responsibility or liability arising from any information contained in or any error of omission from the protocol or from the use of any information contained in it.
